# Gentamicin‐Loaded Carbonate Apatite with Dual Antibacterial and Osteogenic Functions for Combating Surgical Site Infections

**DOI:** 10.1002/adhm.202503739

**Published:** 2025-10-26

**Authors:** Linghao Xiao, Gabriela Laranjeira Abe, Jun‐Ichi Sasaki, Haruaki Kitagawa, Ririko Tsuboi, Tomoki Kohno, Satoshi Imazato

**Affiliations:** ^1^ Joint Research Laboratory of Advanced Functional Materials Science The University of Osaka, Graduate School of Dentistry 1‐8 Yamadaoka Suita Osaka 565‐0871 Japan; ^2^ Department of Dental Biomaterials The University of Osaka, Graduate School of Dentistry 1‐8 Yamada‐Oka Suita Osaka 565‐0871 Japan; ^3^ Department of Cariology, Restorative Sciences and Endodontics University of Michigan School of Dentistry 1011 N University Avenue Ann Arbor MI USA 48104

**Keywords:** antibacterial effects, biomimetic bone substitute, carbonate apatite, gentamicin, neutrophils

## Abstract

Surgical site infections remain a critical concern in dental and orthopedic procedures. To combat surgical site infections, gentamicin‐loaded carbonate apatite (GM‐CAp) granules, a novel antibacterial biomimetic bone substitute is developed. GM‐CAp granules rapidly release the loaded gentamicin, leading to swift suppression of bacteria and proactive prevention of infection. In vitro studies validate the bactericidal effects of GM‐CAp granules against a spectrum of odontogenic bacteria. In vivo investigations confirm the effectiveness of GM‐CAp in suppressing *Staphylococcus aureus* infection, mitigating inflammation, especially neutrophil recruitment, and stimulating bone regeneration. Transcriptomic analysis reveals that early‐stage neutrophil dynamics may influence the inflammatory milieu and subsequent bone healing in the infected bone defect following CAp or GM‐CAp implantation. Overall, GM‐CAp granules represent a potential strategy for combating surgical site infections and increasing the success rate of bone healing in dental and orthopedic surgeries. Additionally, it is proposed that the status of early postoperative neutrophil recruitment may serve as a potential parameter for evaluating the antimicrobial efficacy of biomaterials.

## Graphical abstract

A bone substitute with gentamicin physically precipitated onto the surface of carbonate apatite exhibits prompt drug release, high bactericidal activity, and osteogenic capacity. Efficient antibacterial activity mitigates early postoperative neutrophil accumulation, the status of which may serve as a potential parameter for evaluating the antibacterial efficacy of biomaterials.

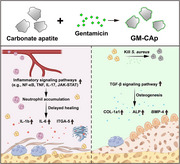

## 1 Introduction

The growing demand for bone reconstruction in dentistry and orthopedics has driven advances in dental implants, periodontal therapies, and bone‐related interventions, although these advances have not significantly reduced the risk of surgical site infections (SSIs).^[^ ^1^, ^2^, ^3^, ^4^ ^]^ SSIs occur in up to 31.2% of oral reconstruction surgeries, imposing a substantial burden on patients and healthcare systems.^[^ ^5^ ^]^ Owing to the oral cavity's bacteria‐rich environment, biomaterial implantation can introduce pathogens, leading to biofilm formation and infection, which in high‐risk dental procedures can cause bacteremia and subsequent infective endocarditis.^[^ ^3^, ^4^, ^6^, ^7^, ^8^ ^]^ To solve this problem, prophylactic systemic antibiotics are commonly prescribed.^[^ ^4^, ^9^ ^]^ However, to achieve minimum inhibitory concentration (MIC) or minimum bactericidal concentration (MBC) at the surgical site, excessive dosing of antibiotics or reducing the dosing interval is often required, which can lead to the emergence of drug‐resistant bacteria and organ damage.^[^ ^10^, ^11^ ^]^

A promising approach to address the above‐mentioned issue is local antibiotic delivery via antibacterial agent‐modified bone substitutes.^[^ ^12^, ^13^, ^14^ ^]^ This strategy aims to achieve high drug concentration at the infection site while minimizing the potential for systemic absorption and toxicity.^[^ ^14^, ^15^, ^16^, ^17^ ^]^ Antibacterial activity is crucial in the early postoperative period because bacteria can rapidly colonize the wound or implant surface and then form a protective biofilm, typically within hours to days.^[^ ^18^, ^19^, ^20^, ^21^ ^]^ Because SSI‐causing bacteria can colonize any part of the surgical area at variable concentrations, surface structural modification or sustained‐release systems alone may not provide sufficiently rapid bactericidal activity.^[^ ^22^ ^]^ Therefore, we designed an antibiotic‐loaded bone substitute material to provide an intentional burst release of antibiotics in the early postoperative period, with the aim of ensuring a rapid antibacterial effect to eliminate contaminates before biofilm formation, thereby reducing the risk of SSIs and subsequently allowing the scaffold to exert its osteogenic function.

Carbonate apatite (CAp, Ca_10‐a_(CO_3_)_b_(PO_4_)_6‐c_) is a bioactive bone substitute with a bone mineral composition similar to that of vertebrate bones, containing 6–9 mass% carbonate.^[^ ^23^, ^24^, ^25^ ^]^ Compared with representative synthetic bone substitutes (such as calcium phosphate ceramics and bioactive glasses), CAp more accurately mimics natural bone in mineral composition and thus mechanical strength and biological properties, enabling the restoration of bone functions.^[^ ^24^, ^26^, ^27^, ^28^, ^29^, ^30^, ^31^ ^]^ Although CAp is thermodynamically stable under physiological pH, it is more soluble than hydroxyapatite in the weakly acidic environments of osteoclasts,^[^ ^24^ ^]^ enabling CAp to participate in bone remodeling. In addition, owing to its pH‐dependent solubility, CAp can be used to selectively release drugs under specific pH conditions (such as acidic pH environments). CAp micropores between apatite crystals enable the absorption and release of antibiotics, exhibiting excellent drug loading efficiency and release profiles to effectively suppress bacterial colonization without compromising bone regeneration.^[^ ^32^, ^33^, ^34^ ^]^ Therefore, CAp is considered an ideal candidate to carry antibacterial agents for local prophylaxis of SSIs.

Gentamicin, an aminoglycoside antibiotic approved by the FDA in 1996, is effective against many aerobic Gram‐negative and some aerobic Gram‐positive organisms. It exhibits broad‐spectrum activity against *Staphylococcus aureus*, *Staphylococcus epidermidis*, and even some antibiotic‐resistant bacterial strains.^[^ ^35^ ^]^ This broad‐spectrum antibiotic acts by irreversibly binding to ribosomal subunits to inhibit bacterial protein synthesis, effectively treating osteomyelitis.^[^ ^36^, ^37^ ^]^ Compared with natural (e.g., tea polyphenols),^[^ ^38^ ^]^ synthetic (e.g., phenolic compounds),^[^ ^39^ ^]^ and inorganic (e.g., silver ions)^[^ ^40^ ^]^ antibacterial agents, gentamicin offers broad‐spectrum bactericidal activity against a wide range of pathogens, with a proven clinical track record, making it a biosafe and highly effective antimicrobial agent for combating SSIs.^[^ ^41^, ^42^ ^]^

In this study, gentamicin‐loaded carbonate apatite (GM‐CAp) granules were developed to release broad‐spectrum antibiotic gentamicin from the surface of CAp granules in the early stage of SSIs. The hypothesis was that these novel GM‐CAp granules would combat bacterial colonization and proliferation at the surgical site during the early stage of bone remodeling through gentamicin release and subsequently exert their osteoconductivity, achieving stepwise treatment. The in vitro biocompatibility and antibacterial effects of the granules were assessed using primary human bone marrow‐derived mesenchymal stem cells (MSCs) and odontogenic bacteria. Subsequently, the effects of GM‐CAp granules on bacterial growth, immune responses, and bone tissue regeneration were evaluated using in vivo assays.

## 2 Results

### 2.1 Gentamicin Release from GM‐CAp

**Figure 1:**
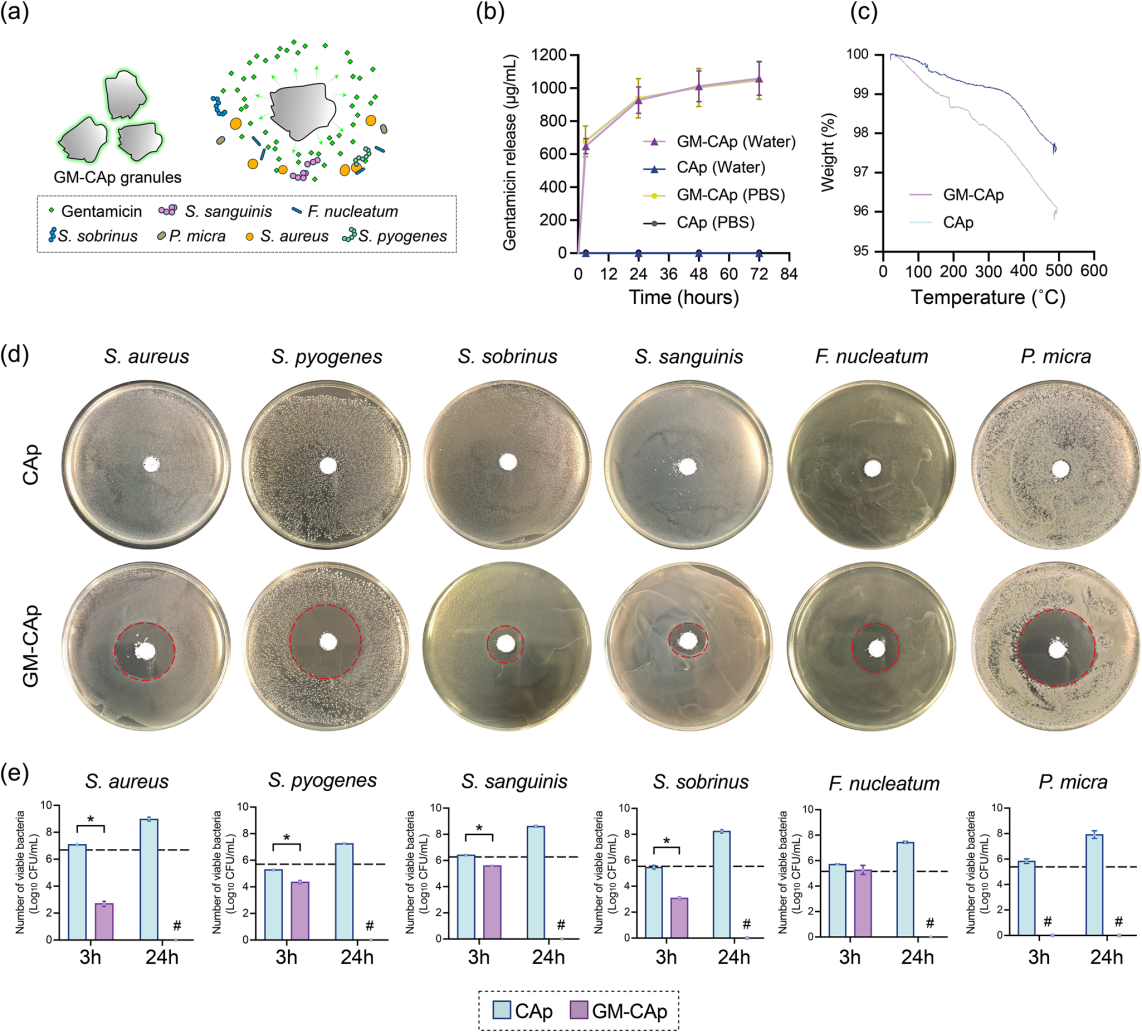
Release profile and in vitro antibacterial effect of GM‐CAp granules against odontogenic bacteria. a) Schematic representation of the in vitro antibacterial effect of GM‐CAp granules. b) Release of gentamicin from CAp and GM‐CAp granules within 72 h in distilled water and PBS (*n* = 3). c) TG‐DTA of CAp and GM‐CAp granules heated up to 500 °C under air (*n* = 3). d) Inhibition zones of CAp and GM‐CAP granules incubated with six odontogenic bacteria (*S. aureus*, *S. pyogenes*, *S. sanguinis*, *S. sobrinus*, *F. nucleatum*, and *P. micra*) for 24 h (*n* = 3). e) Number of viable bacteria following the incubation of six odontogenic bacteria with CAp and GM‐CAp granules for 3 and 24 h (*n* = 3). Dashed line indicates initial bacterial number. # indicates no detected bacteria. * *p* < 0.05.

The concentration of the gentamicin sulfate solution used to load gentamicin onto CAp granules was adjusted according to the 72 h release profile of gentamicin from the resulting GM‐CAp granules into distilled water, as shown in Figure S1 (Supporting Information). When the concentration of the gentamicin sulfate solution was 200 mg mL^−1^, the amount of gentamicin released from the resulting GM‐CAp granules was 63.5% of that released from a commercially available gentamicin‐loaded bone cement, G‐HV (Cobalt G‐HV Bone Cement, Enovis, Austin, USA), under the same conditions. On this basis, the 200 mg mL^−1^ gentamicin sulfate solution was selected for material preparation. **Figure** **1**a shows a schematic representation of the in vitro antibacterial effect of GM‐CAp capsules. Figure 1b illustrates the release profiles of gentamicin from GM‐CAp and CAp granules in distilled water and PBS. GM‐CAp exhibited similar drug release profiles under both conditions. In distilled water, a burst release of gentamicin from GM‐CAp granules was observed within the first 3 h. Initially, 50.26% of loaded gentamicin was released from the GM‐CAp granules. Subsequently, the release rate decreased, plateauing at 48–72 h. By 72 h, 82.08% of gentamicin was released from the GM‐CAp granules. No gentamicin release was detected from CAp granules under either condition. The release profiles of gentamicin from GM‐CAp granules and commercial G‐HV were compared, as shown in Figure S2 (Supporting Information). There was no significant difference in gentamicin release between the two materials within the first 48 h in distilled water, although gentamicin release from GM‐CAp granules was significantly lower than that from G‐HV after 48 h. Thermogravimetry‐differential thermal analysis (TG‐DTA) showed that the gentamicin content of GM‐CAp granules was 1.29 ± 0.39 wt.% (Figure 1c).

### 2.2 MIC and MBC of Gentamicin Sulfate Against Six Oral Bacterial Species

The MIC of gentamicin sulfate against 6 oral bacteria, namely *S. aureus*, *Streptococcus pyogenes*, *Streptococcus sanguinis*, *Streptococcus sobrinus*, *Fusobacterium nucleatum*, and *Parvimonas micra*, ranged from 8 to 128 µg mL^−1^, and the MBC ranged from 16 to 128 µg mL^−1^ (**Table** **1**).

**Table 1:** MIC and MBC of gentamicin sulfate against 6 oral bacteria.

Species	MIC (µg mL^−1^)	MBC (µg mL^−1^)
*Staphylococcus aureus*	32	64
*Streptococcus pyogenes*	8	16
*Streptococcus sanguinis*	128	128
*Streptococcus sobrinus*	64	64
*Fusobacterium nucleatum*	128	128
*Parvimonas micra*	32	32

### 2.3 In Vitro Antibacterial Effects of GM‐CAp Granules

GM‐CAp granules produced inhibition zones against all six tested oral bacterial species (Figure 1d). In contrast, CAp granules did not inhibit the growth of these six bacterial species. Figure 1e shows the number of viable bacteria after the bacteria were incubated with CAp or GM‐CAp granules. After 3 h of incubation, GM‐CAp significantly reduced the viability of 5 of the bacterial species, other than *F. nucleatum*, and completely eliminated *P. micra*. After 24 h of incubation, GM‐CAp eradicated all 6 bacterial species. In contrast, CAp granules did not inhibit bacterial growth, and all six species exhibited normal growth patterns.

### 2.4 In Vitro Biocompatibility of GM‐CAp Granules

**Figure 2:**
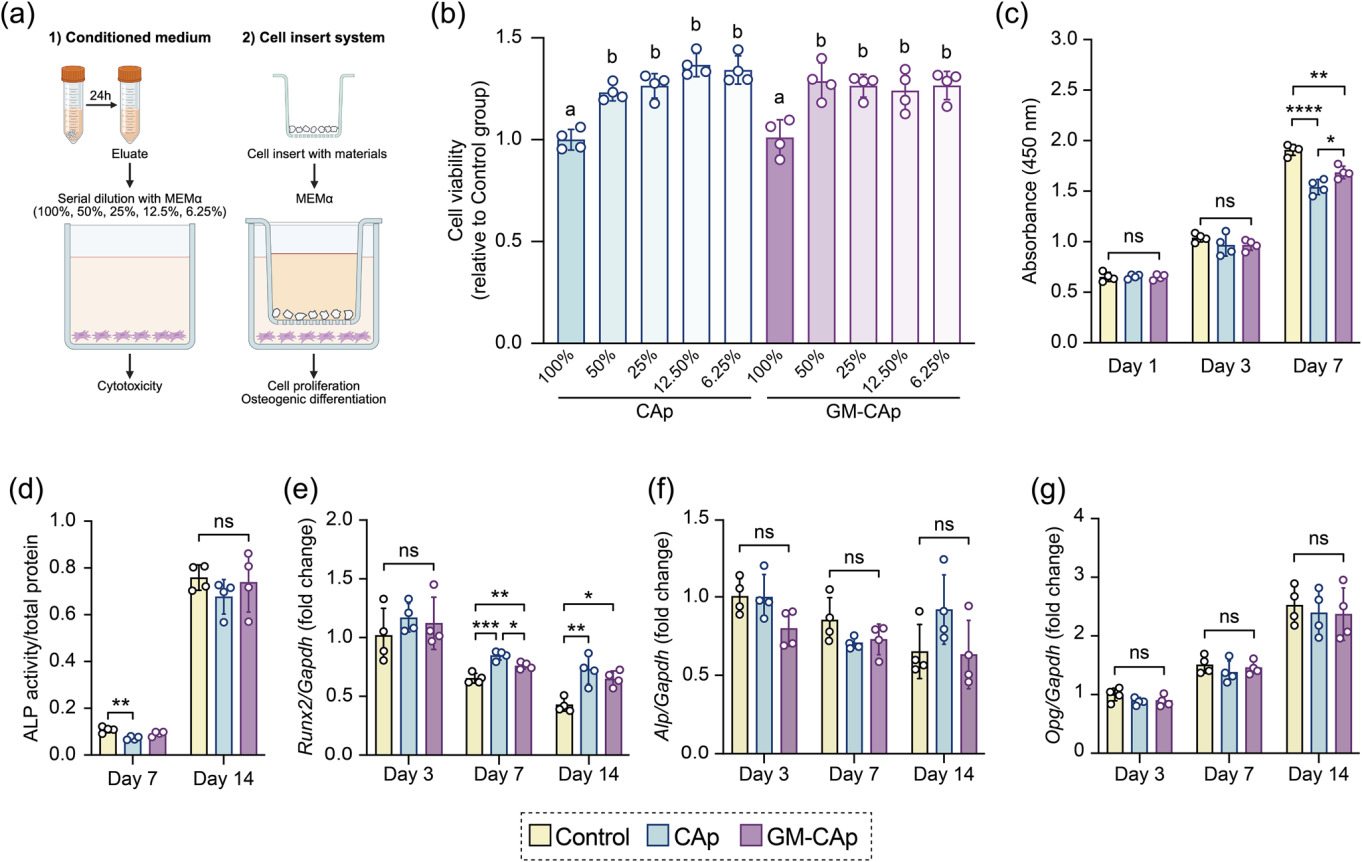
Impact of CAp and GM‐CAp granules on human‐derived MSCs. a) Schema of cell culture method, including the conditioned medium and culture insert system. b) Cell viability after 24 h of culturing with CAp and GM‐CAp eluates. Different letters indicate significant statistical differences (*p <* 0.01) (*n* = 4). c) Proliferation of MSCs after 1, 3, and 7 days of culturing with CAp and GM‐CAp granules (*n* = 4). “ns” indicates no significant difference among three groups. * *p* < 0.05, ** *p* < 0.01, **** *p* < 0.0001. d) ALP activity of MSCs after 7 and 14 days of culturing with CAp and GM‐CAp granules (*n* = 4). “ns” indicates no significant difference among three groups. ** *p* < 0.01. e–g) Osteogenic gene expression profiles (*Runx2*, *Alp*, and *Opg*) of MSCs after 3, 7, and 14 days of culturing with CAp and GM‐CAp granules (*n* = 4). “ns” indicates no significant difference among three groups. * *p* < 0.05, ** *p* < 0.01, *** *p* < 0.001.

**Figure** **2** illustrates the impact of CAp and GM‐CAp granules on human‐derived MSCs. Figure 2a shows the schema of the cell culture method, including the conditioned medium and cell insert system. The cytotoxicity assay revealed no significant difference in the viability of cells treated with 100% eluate from CAp granules and those treated with 100% eluate from GM‐CAp granules (Figure 2b). However, cell viability increased as the eluate concentration decreased in a dose‐dependent manner. A dose‐response study was conducted to determine the optimal treatment dose of CAp and GM‐CAp granules (Figure S3, Supporting Information). Compared with the control group, cells cultured with 20 mg of CAp granules for 7 days exhibited significantly reduced viability. On the basis of these results, 10 mg was selected as the treatment dose for subsequent cell experiments.

The cell proliferation assay demonstrated that MSCs cultured with CAp or GM‐CAp granules for 7 days exhibited proliferative activity (Figure 2c). After 7 days, the cell number was significantly higher in the GM‐CAp group than in the CAp group. The influence of GM‐CAp granules on osteogenic differentiation was assessed by measuring alkaline phosphatase (ALP) activity (Figure 2d) and analyzing osteogenic marker expression (Figure 2e–g). Throughout 14 days of culturing, gentamicin release did not affect ALP activity in MSCs (Figure 2d). After 7 days of culturing, runt‐related transcription factor 2 (*Runx2)* gene expression in cells cultured with CAp or GM‐CAp granules was significantly upregulated compared with that in the control group (Figure 2e). However, the expression of alkaline phosphatase (*Alp)* and osteoprotegerin (*Opg*) genes remained unaffected by either type of granules.

### 2.5 In Vivo Antibacterial And Osteogenic Functions of GM‐CAp Granules

#### 2.5.1 Clinical Symptoms

**Figure 3:**
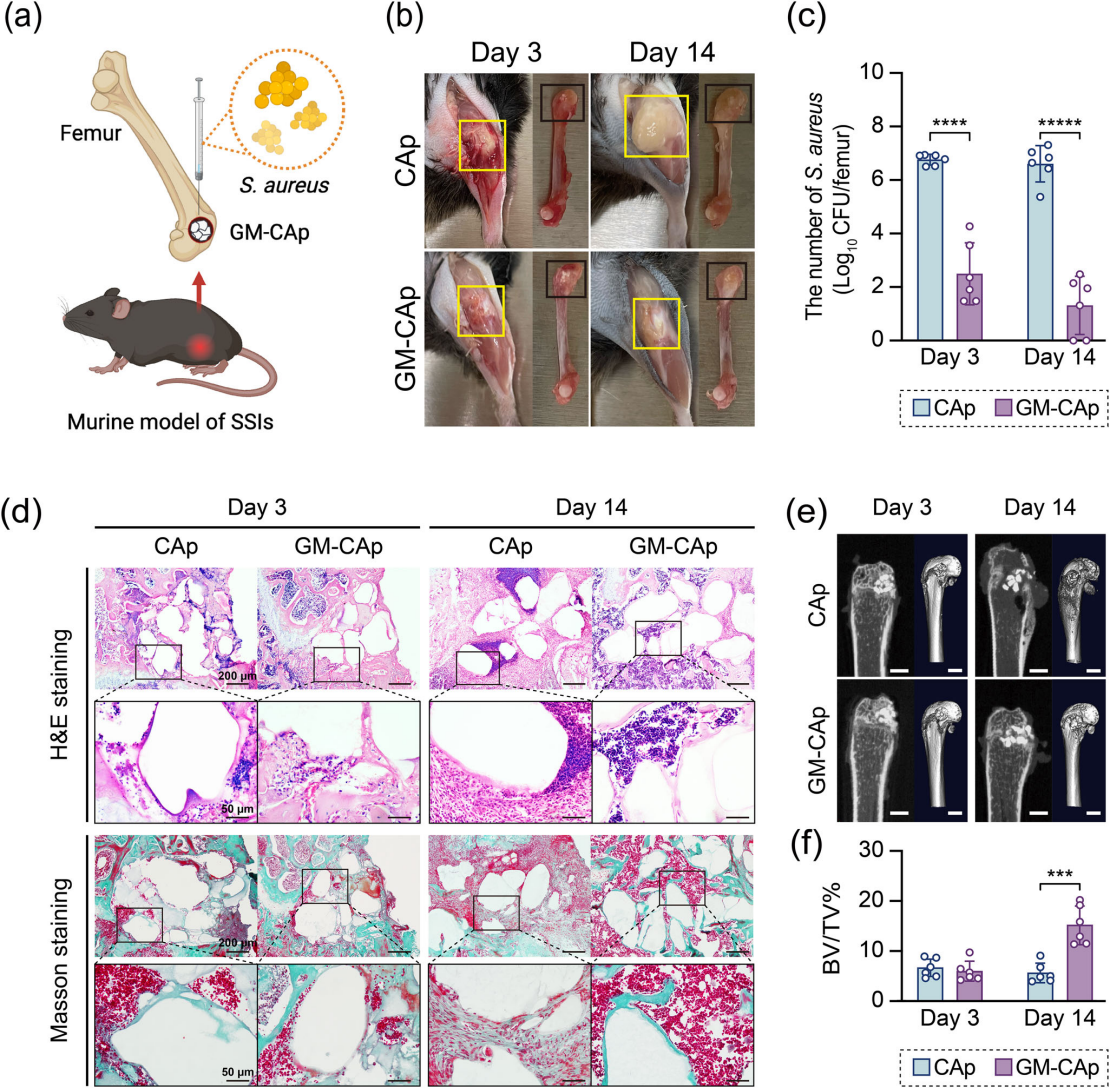
In vivo antibacterial effect of CAp and GM‐CAp granules in C57BL/6JJcl mice. a) Design of the murine model of bone infection. b) Photographs of surgical sites and femurs in CAp and GM‐CAp groups at 3 and 14 days post‐surgery. The yellow frame indicates the surgical sites and covered soft tissues. The black frame indicates the bone defects. c) Bacterial burden in CAp and GM‐CAp groups at 3 and 14 days post‐surgery (*n* = 6). **** *p* < 0.0001, ***** *p* < 0.00001. d) H&E and Masson–Goldner staining of tissues in CAp and GM‐CAp groups at 3 and 14 days post‐surgery. e) Micro‐CT images of tissues around surgical sites in CAp and GM‐CAp groups. Scale bars = 1 mm. f) Ratio of bone volume to tissue volume (BV/TV%) at 3 and 14 days post‐surgery (*n* = 6). *** *p* < 0.001.

The design of the murine model of SSIs is shown in **Figure** **3**a. At 3 days post‐surgery, the surgical site of mice in the CAp group exhibited redness, swelling, and abscess formation. In contrast, mice in the GM‐CAp group displayed only mild congestion without abscess formation (Figure 3b). At 14 days post‐surgery, mice in the CAp group showed significant abscess accumulation and bone tissue whitening, indicative of tissue ischemia. Mice in the GM‐CAp group, however, exhibited healed soft tissues without noticeable swelling or abscess formation (Figure 3b). A detailed summary of the clinical symptoms of mice in each group at each time point is provided in Table S1 (Supporting Information).

#### 2.5.2 Bacterial Burden

*S. aureus* in the femur was quantified in terms of colony‐forming units (CFU) (Figure 3c). The count of *S. aureus* was converted from CFU/femur to log_10_ CFU/femur. At 3 days post‐surgery, the count of *S. aureus* in the CAp group exhibited a 158.50‐fold increase, from the initial inoculation amount of 4.56 log_10_ CFU/femur to 6.76 ± 0.20 log_10_ CFU per femur. In contrast, the count of *S. aureus* in the GM‐CAp group exhibited a significant reduction to 2.50 ± 1.16 log_10_ CFU per femur, which was 8.7% of the initial inoculation amount and 0.005% of the CAp group. At 14 days post‐surgery, the count of *S. aureus* in the CAp group decreased slightly to 6.61 ± 0.68 log_10_ CFU per femur, which was similar to that at 3 days post‐surgery, while that in the GM‐CAp group decreased to 1.31 ± 1.09 log_10_ CFU per femur, which was 0.056% of the initial inoculation amount and 0.0005% of the CAp group.

#### 2.5.3 Radiographic Findings

Micro‐computed tomography (CT) images (Figure 3e) revealed no significant low‐density lesions around the surgical site of mice in either group at 3 days post‐surgery. However, at 14 days post‐surgery, mice in the CAp group exhibited extensive bone destruction, including sparse or absent trabecular bone at the proximal and distal ends of the defects, thinned cortical bone, and disrupted cortical bone continuity. Moreover, mice in the CAp group exhibited osteolytic lesions. In contrast, mice in the GM‐CAp group maintained a dense bone structure at the proximal and distal ends of the defect, with no significant changes up to 14 days post‐surgery. Trabecular bone was evenly distributed throughout the bone marrow, and the cortical bone structure remained intact, except in the defect area. The ratio of bone volume to tissue volume (BV/TV%) indicated that there was no significant difference in bone formation at 3 days post‐surgery between CAp and GM‐CAp groups (Figure 3f). By 14 days post‐surgery, distinct bone regenerated in areas surrounding the granules of GM‐CAp, and the volume ratio of newly formed bone to tissue volume was significantly higher in the GM‐CAp group than in the CAp group.

#### 2.5.4 Histopathological Analysis

At 3 days post‐surgery, hematoxylin and eosin (H&E) staining of tissues in the CAp group revealed signs of acute bone infection, including a high level of immune cell infiltration and a large amount of necrotic cell debris around the CAp granules (Figure 3d). At 14 days post‐surgery, the CAp group exhibited extensive bone destruction, trabecular bone resorption, and fibrous tissue formation. Tertiary lymphoid structure was observed around the CAp granules. Organized aggregates of immune cells were found between neighboring CAp granules, which were surrounded by dense granulation tissue, suggesting chronic infection. In contrast, the GM‐CAp group showed bone marrow‐like tissues and new bone formation around the GM‐CAp granules.

Masson–Goldner staining (Figure 3d) revealed marked congestion at the surgical site in both groups at 3 days post‐surgery. At 14 days post‐surgery, extensive, green‐stained collagen fibers were observed around the CAp granules, indicating ongoing fibrosis in the CAp group. In the GM‐CAp group, green‐stained new bone was observed from the margin of the GM‐CAp granules, with less fibrosis. These findings indicated that GM‐CAp granules suppressed acute chronic inflammation caused by *S. aureus* infection, promoting a favorable environment for bone healing and regeneration.

**Figure 4:**
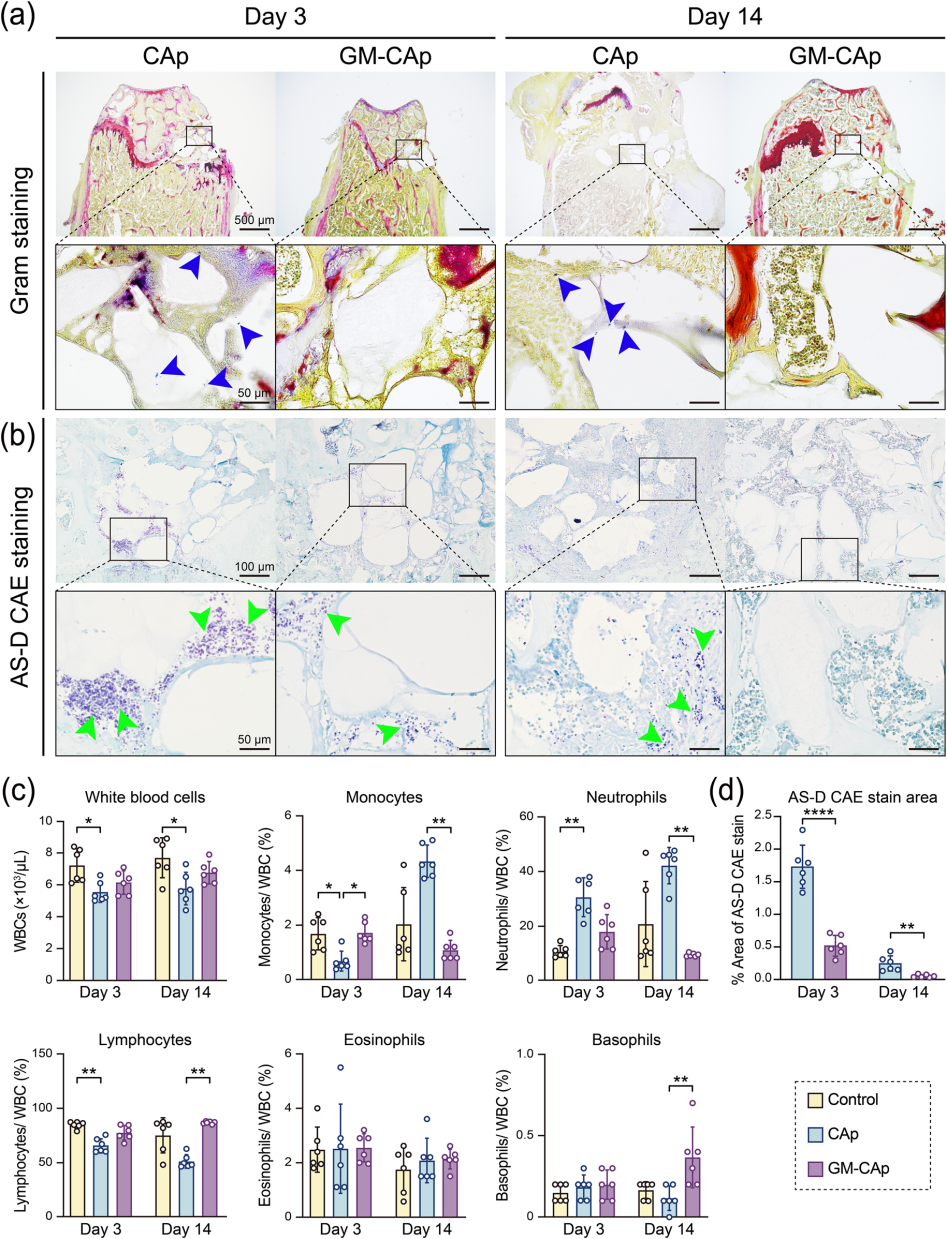
Histopathological and hematological analyses. a) Gram and b) AS‐D CAE staining of tissues in CAp and GM‐CAp groups at 3 and 14 days post‐surgery. Blue arrowheads point to identified *S. aureus* colonies, and green arrowheads point to positive stained neutrophils. c) Hematological analysis at 3 and 14 days post‐surgery to evaluate total WBC counts and percentages of specific types of WBCs, namely monocytes, neutrophils, lymphocytes, eosinophils, and basophils (*n* = 6). The control group is composed of healthy mice without intervention. d) Quantitative analysis of stained tissues: percentage of AS‐D CAE stained area relative to the total defect area (*n* = 6). * *p* < 0.05, ** *p* < 0.01, **** *p* < 0.0001.

Gram staining (modified Brown & Brenn) of tissues in both groups enabled the detection of viable *S. aureus* colonies around CAp and GM‐CAp granules at 3 and 14 days post‐surgery (**Figure** **4**a). Blue‐stained *S. aureus* colonies were observed in the CAp group at 3 and 14 days post‐surgery (marked by the blue arrowheads), which was consistent with the bacterial burden results (Figure 3c). In the CAp group, *S. aureus* colonies were found around the CAp granules, which also spread to the distal end of the surgical site at 14 days post‐surgery. However, less bacteria were found in the GM‐CAp group at 3 and 14 days post‐surgery, suggesting the clearance of most of the *S. aureus* colonies.

AS‐D chloroacetate esterase (CAE) staining was performed to assess neutrophil infiltration in response to SSIs (Figure 4b). We observed a large number of stained neutrophils in the area of the implanted material. These cells tended to be located around the implanted material. Figure 4d shows the percentage of AS‐D CAE‐stained area relative to the total defect area, which was determined from quantitative analysis of stained tissues. Compared with the CAp group, the GM‐CAp group showed significantly reduced neutrophil infiltration around the granules on day 3 (marked by green arrowheads). By day 14, there was almost no residual neutrophil infiltration in the GM‐CAp group, whereas scattered neutrophils persisted in the CAp group, suggesting ongoing mild bacterial inflammation. These findings strongly suggested that the GM‐CAp granules exerted a local antibacterial effect.

#### 2.5.5 Hematological Analysis

Hematological analysis (Figure 4c) revealed that the count of WBCs in the CAp group was slightly lower than that in the control group, while the count of WBCs in the GM‐CAp group remained at a similar level to that in the control group. The control group was composed of age‐matched healthy mice without intervention, and the results for this group could be considered as representing normal hematological levels. For each parameter, 95% confidence intervals (CIs) of the value within 3‐ and 14‐day groups were calculated to indicate the precision of the estimated means (Table S2, Supporting Information). In the CAp group, the significantly increased neutrophil level, compared with that in the control, persisted from 3 days post‐surgery to 14 days post‐surgery, indicating ongoing inflammation and osteomyelitis. In contrast, the neutrophil level in the GM‐CAp group remained similar to that in the control group, suggesting the absence of osteomyelitis. In the CAp group, the monocyte ratio decreased at 3 days post‐surgery and increased significantly at 14 days post‐surgery compared with that in the control, indicating chronic bacterial infection. In contrast, the monocyte level in the GM‐CAp group remained at a similar level to that in the control group, suggesting the suppression of bacterial infection and chronic inflammation for 14 days post‐surgery. The lymphocyte level in the CAp group decreased compared with that in the control owing to the increase in neutrophil and monocyte levels. The eosinophil levels in the CAp and GM‐CAp groups were similar to that in the control group, suggesting that the eosinophil level was unaffected by bacterial inoculation or material implantation. The basophil level in the GM‐CAp group was significantly higher than that in the CAp group at 14 days post‐surgery. However, the difference was relatively small compared with that of other leukocyte subsets. Supplementary hematological data are summarized in Figure S4 (Supporting Information).

#### 2.5.6 Gene Expression and Bioinformatic Analysis

**Figure 5:**
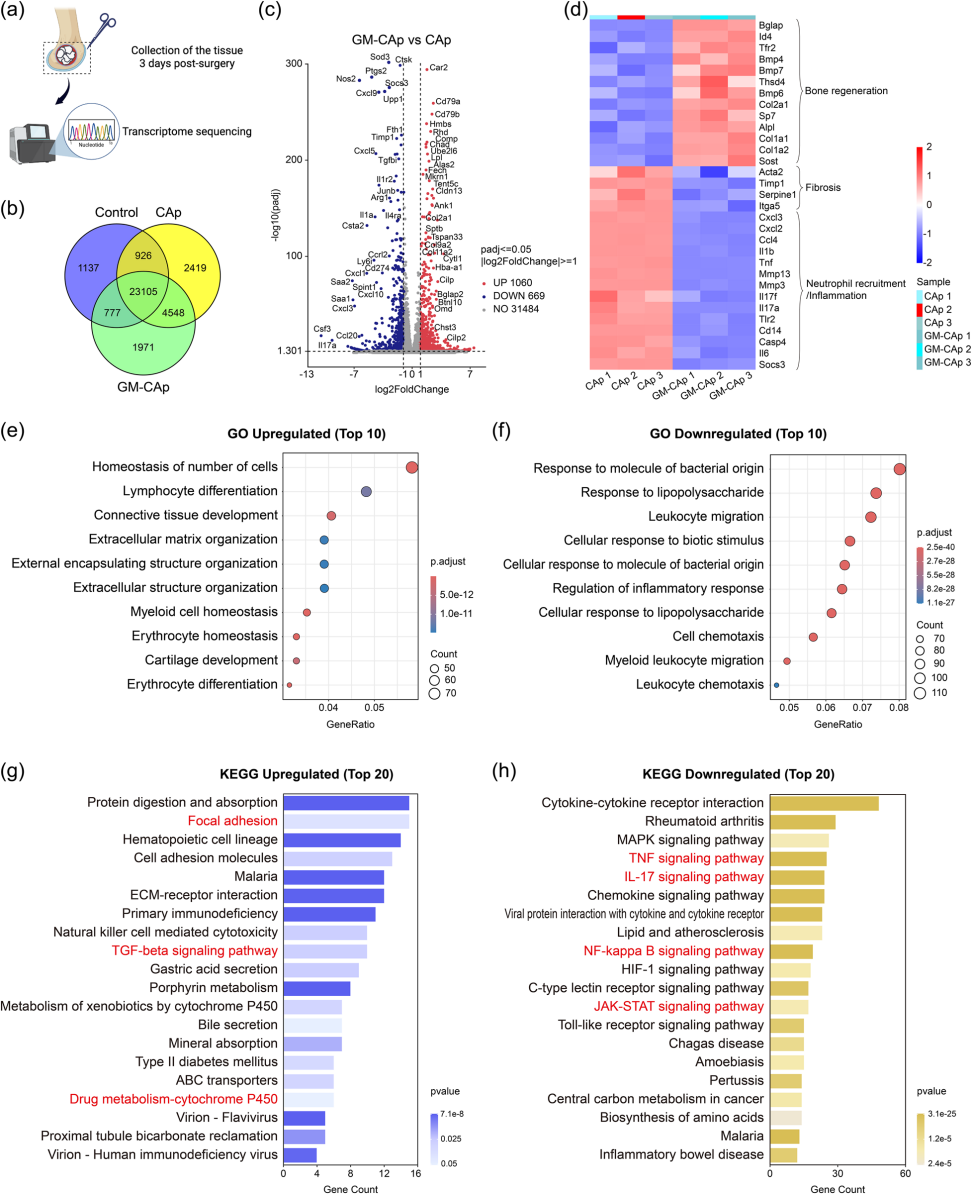
Gene expression and bioinformatic analysis. a) Schematic illustration of postoperative tissue extraction for transcriptomic sequencing. b) Venn diagram of co‐expression among control, CAp, and GM‐CAp groups. The control group is composed of healthy mice without intervention. c) Volcano map of differential genes between CAp and GM‐Cap groups. The most significantly upregulated and downregulated genes are annotated. d) Heat map of interesting genes related to bone regeneration, fibrosis, and neutrophil recruitment/inflammation. e,f) GO analysis of GM‐CAp group, including the top 10 upregulated and top 10 downregulated biological processes. g,h) KEGG pathway analysis of GM‐CAp group, including the top 20 upregulated and top 20 downregulated pathways.

In the early stages of SSIs, the antibacterial properties of the implanted material directly influence innate immune responses, significantly impacting bone healing. To investigate the underlying mechanisms, RNA sequencing was performed on infected bone tissues at 3 days post‐surgery (**Figure** **5**a). The control group was composed of age‐matched healthy mice without intervention. Gene expression in the control group could be considered as gene levels in the healthy state. A total of 34883 genes were detected across the three groups. As shown in the Venn diagram (Figure 5b), 89.2% of genes in the CAp group and 90.9% of genes in the GM‐CAp group were co‐expressed. Differential expression analysis identified 1729 differentially expressed genes (DEGs), including 1016 upregulated and 669 downregulated genes in the GM‐CAp group, compared with those in the CAp group (Figure 5c). The most significantly upregulated and downregulated genes are annotated. Genes related to the TGF‐β signaling pathway, such as *Comp*, *Chad*, *Col2a1*, *Col9a2*, *Cilp*, *Cilp2*, *Chst3*, *Omd*, *and Bglap2*, were significantly upregulated in the GM‐CAp group compared with those in the CAp group. These genes contribute to extracellular matrix formation, osteogenic differentiation, and tissue remodeling. Moreover, genes such as *Ptgs2*, *Nos2*, *Cxcl1*, *Cxcl5*, *Cxcl9*, *Cxcl10*, *Ccl20*, *Saa1*, *Saa2*, *Csf3*, *Il1a*, *Il1r2*, *Junb*, *Socs3*, *Arg1*, *Il4ra*, and *Cd274* were significantly downregulated in GM‐CAp group compared with those in the CAp group. These genes are directly related to inflammatory signaling pathways, including NF‐κB, TNF, IL‐17, and JAK‐STAT signaling pathways, thereby inducing chronic inflammation and delaying healing. Hierarchical clustering revealed distinct expression patterns, as visualized in the heat map (Figure 5d). In the GM‐CAp group, genes related to bone regeneration, such as *Bmp4*, *Col2a1*, and *Alpl*, were significantly upregulated. In contrast, genes associated with chronic inflammation and fibrosis (e.g., *Acta2*, *Timp1*, and *Itga5*) as well as pro‐inflammatory chemokines (e.g., *Cxcl3*, *Ccl4*, and *Il1b*) were downregulated.

Gene Ontology (GO) enrichment analysis demonstrated that GM‐CAp significantly upregulated immune‐related biological processes, including cell count homeostasis, myeloid cell homeostasis, lymphocyte differentiation, and erythrocyte homeostasis and differentiation, as well as tissue remodeling‐related biological processes such as connective tissue development, extracellular matrix organization, extracellular structure organization, and cartilage development (Figure 5e). These findings were consistent with the above histological evidence of new bone formation and the quantification of neutrophils in both blood and tissues. Conversely, GM‐CAp markedly downregulated biological processes related to bacterial responses (e.g., response to lipopolysaccharides and response to molecules of bacterial origin), inflammatory signaling (e.g., regulation of inflammatory response), and chemotaxis (e.g., cell chemotaxis, myeloid leukocyte migration) (Figure 5f). These findings indicated that GM‐CAp contributed to immune environment homeostasis, especially the avoidance of excessive neutrophilic infiltration.

Pathway analysis using the Kyoto Encyclopedia of Genes and Genomes (KEGG) revealed that GM‐CAp significantly upregulated pathways associated with tissue regeneration, including focal adhesion and TGF‐beta signaling pathways, as well as pathways related to drug metabolism such as cytochrome P450‐mediated drug metabolism (Figure 5g). In contrast, several inflammation‐related pathways, including TNF signaling, IL‐17 signaling, NF‐κB signaling, and JAK‐STAT signaling, were notably downregulated (Figure 5h). Additionally, we mapped all neutrophil extracellular trap (NET) formation signaling pathway‐associated transcripts onto the KEGG pathway (Figure S5, Supporting Information) and identified a total of 13 affected transcripts (7 upregulated, 6 downregulated). These results suggested that gentamicin released from GM‐CAp likely underwent host metabolic processing while contributing to effective infection control. The concurrent suppression of pro‐inflammatory signaling pathways suggests a transition from acute inflammation to immune resolution and tissue repair.

## 3 Discussion

The present study successfully synthesized GM‐CAp granules with prompt drug release. Gentamicin was physically precipitated onto the surface of CAp granules, which significantly accelerated its release. The material properties of the CAp granules (Cytrans, GC, Japan) used in this study have been well characterized in previous studies, showing a dense microstructure and high bulk density that confer superior mechanical strength compared with those of HA and β‐TCP granules.^[^ ^43^ ^]^ Because gentamicin was deposited on the surface of CAp granules and rapidly eluted, it is unlikely to have significantly altered the intrinsic porosity, surface area, or mechanical integrity of the CAp matrix. Compared with previously reported gentamicin‐loaded biomaterials, which release over 90% of the encapsulated drug from 140 h to 30 days,^[^ ^44^, ^45^, ^46^, ^47^, ^48^ ^]^ GM‐CAp exhibited faster drug release in vitro, with 82.08% of gentamicin released within 72 h in distilled water. It should be noted that one of the cited studies^[^ ^48^ ^]^ reported clinical data on the local gentamicin concentration of patients, which are not directly comparable with present experimental results. The release profiles observed under the ideal conditions of distilled water were similar to those in PBS, indicating that drug release was minimally affected by the ionic environment. This prompt release overcomes the shortcoming of prolonged antibiotic administration, which not only fails to reduce SSIs, but also poses the risk of adverse effects, bacteria resistance, or both.^[^ ^8^, ^49^ ^]^ The conditions for preparing GM‐CAp were optimized on the basis of the gentamicin release characteristics of G‐HV, a setting poly (methyl methacrylate)‐based bone cement used in two‐stage revision surgeries. However, unlike G‐HV, GM‐CAp exhibits osteoconductivity owing to its carbonate apatite scaffold. CAp closely mimics the mineral component of natural bone, enabling it to participate in physiological bone remodeling.^[^ ^24^ ^]^ Osteoclasts actively resorb CAp, which in turn stimulates osteoblast differentiation and activity, thereby promoting new bone formation.^[^ ^50^ ^]^ This osteoclast‐mediated resorption mirrors the natural bone turnover cycle, allowing CAp to be gradually replaced by mature bone. Additionally, the porous structure of GM‐CAp facilitates cellular infiltration, vascularization, and overall bone ingrowth and maturation.^[^ ^32^ ^]^ The release profile was further compared with the MIC and MBC of gentamicin sulfate. Within the first 24 h, the concentration of released gentamicin was higher than the MIC and MBC of gentamicin sulfate against 6 oral bacterial species, indicating that GM‐CAp granules eliminated all 6 bacteria within 24 h, consistent with the in vitro antibacterial effect. After 24 h, the concentration of released gentamicin was equivalent to or lower than the MIC and MBC of gentamicin sulfate against the bacteria, indicating its potential low cytotoxicity.

In vitro studies confirmed the good biocompatibility of GM‐CAp granules with primary human MSCs. Although both CAp and GM‐CAp granules exhibited an inhibitory effect on MSCs 7 days after treatment (Figure 2b,c), likely because carbonate apatite adsorbed proteins from the culture medium,^[^ ^51^, ^52^, ^53^ ^]^ more than 80% of MSCs remained viable after they were treated with either CAp or GM‐CAp granules, indicating low cytotoxicity within an acceptable range. Importantly, GM‐CAp granules did not further decrease cell viability compared with CAp (Figure 2c), and they did not significantly inhibit ALP activity or osteogenic gene expression (Figure 2d–g). These findings suggest that incorporating gentamicin into CAp does not adversely affect the viability and osteogenic potential of MSCs within 14 days. However, it should be noted that high doses of gentamicin have been reported to reduce cell viability and ALP activity in vitro, potentially impairing bone healing and repair in vivo.^[^ ^54^ ^]^ Conversely, some studies have indicated that local administration of antibiotics around implants does not adversely affect bone formation and might even slightly enhance osseointegration.^[^ ^55^ ^]^ Therefore, further studies are required to clarify the dose‐dependent effects of gentamicin on bone‐forming cells and to identify an optimal therapeutic window for safe and effective local delivery.

Moreover, GM‐CAp granules demonstrated excellent antibacterial activity against SSI‐associated bacteria both in vitro and in vivo. During oral bone reconstruction surgery, despite the application of preoperative disinfection protocols, it is challenging to eliminate resident oral bacteria derived from saliva, the dorsum of the tongue, gingival crevicular fluid, dental plaque, and other intraoral sources. Dental surgeries, such as apicoectomy and alveolar bone augmentation surgery, are often accompanied by bacterial infections.^[^ ^56^, ^57^, ^58^, ^59^, ^60^ ^]^ Odontogenic bacterial infection can spread to the alveolar bone, leading to acute purulent bacterial osteomyelitis.^[^ ^59^, ^60^ ^]^ In vitro, GM‐CAp exhibited excellent bactericidal effect against a variety of odontogenic bacteria, including opportunistic pathogens of odontogenic bacteremia (*S. sanguinis* and *S. sobrinus*) and pathogens of severe dental infections (*S. sobrinus*, *F. nucleatum*, and *P. micra*). In vivo, GM‐CAp granules significantly reduced the time required to control intra‐bone infection, from several weeks post‐surgery (reported as 2–8 weeks)^[^ ^61^, ^62^, ^63^ ^]^ to 3 days (Figure 3c). This effect is attributed to the prompt release of gentamicin, which inhibits early bacterial biofilm formation.^[^ ^64^, ^65^ ^]^

The immunomodulatory effects of GM‐CAp were also evident. In the CAp group, *S. aureus* infection led to a progressive increase in the level of neutrophils at both the periphery and local infection site, indicating activation of the first line of defense of the innate immune system.^[^ ^66^ ^]^ Later, monocytes accumulated in peripheral blood (Figure 4c), with fibrous tissue forming around the CAp granules (Figure 3d), indicating a switch from acute to chronic bone tissue infection.^[^ ^67^ ^]^ In contrast, GM‐CAp granules effectively inhibited excessive neutrophil infiltration, prevented blood disorders, and promoted new bone formation on the surface of the granules, suggesting a timely transition from infection‐related inflammation to bone healing and remodeling. It is worth noting that the increased counts of phagocytes (e.g., monocytes and neutrophils.) in the CAp group compared with those in the control suggest a plausible breaching of locally inoculated bacteria into the circulatory system, reflecting systemic infiltration of the infection. Therefore, the decreased phagocyte counts in the GM‐CAp group compared with those in the control may reflect a direct local immunomodulatory effect of released gentamicin and could also be explained by the prevention of bacteremia in the circulation. Taken together, these findings highlight the dual functionality of GM‐CAp in antibacterial activity and bone regeneration, with potential positive effects on both local inflammation and systemic infection burden.

**Figure 6:**
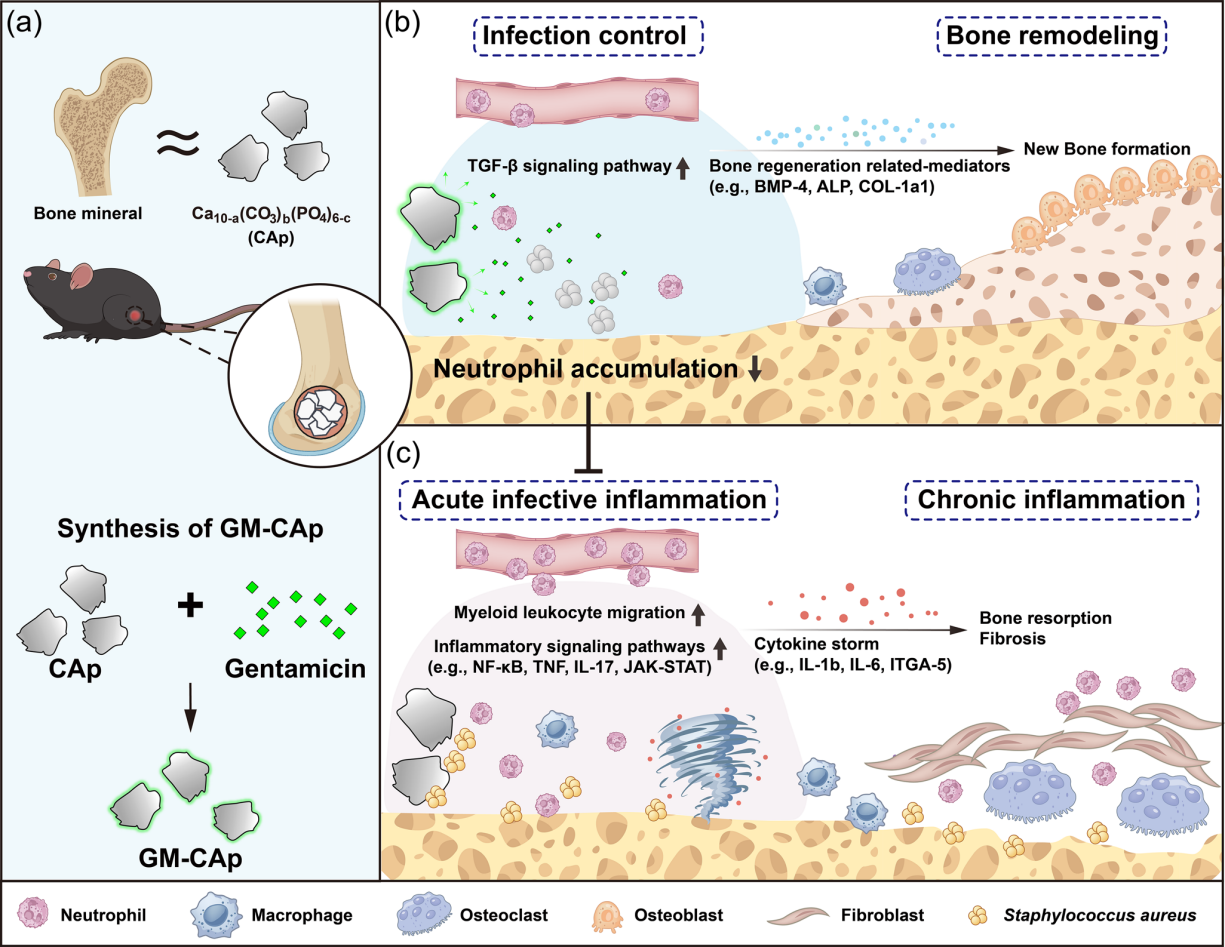
Schematic illustration of the antibacterial and osteogenic effects of GM‐CAp granules. a) GM‐CAp granules were synthesized by physically precipitating gentamicin sulfate onto the surface of CAp granules with a composition closely resembling that of bone mineral. GM‐CAp granules were applied in a murine model of *S. aureus*‐infected bone defects. b) GM‐CAp implantation achieves infection control, mitigating excessive neutrophil accumulation at the early stage of SSIs, accompanied by the suppression of pro‐inflammatory signaling pathways, promoting a transition from acute infection to bone remodeling. c) In the absence of effective infection control, acute infection‐related inflammation leads to persistent neutrophil infiltration and a cytokine storm (e.g., IL‐1b, IL‐6, ITGA‐5), contributing to chronic inflammation, bone resorption, and fibrosis.

Omics analyses further revealed key immunological differences between CAp and GM‐CAp granules during the early stage of SSIs (**Figure** **6**), despite the absence of significant differences in histological, radiographic, or clinical symptoms at 3 days post‐surgery. In the GM‐CAp group, multiple inflammatory pathways were significantly suppressed, suggesting that local gentamicin release effectively eliminated the bacterial infection and attenuated downstream immune activation. Beyond infection control, the immunomodulatory effects of GM‐CAp were evident in the reduced recruitment of neutrophils, likely mediated through suppressed inflammatory‐related signaling pathways. The downregulation of inflammatory signaling pathways such as NF‐κB, TNF, IL‐17, and JAK‐STAT pathways was accompanied by a marked suppression of several chemokine‐related genes, including *Cxcl1*, *Cxcl5*, and *Ccl20* (Figure 5). These chemokines are well‐known downstream effectors that mediate neutrophil chemotaxis via the Cxcr2 receptor axis.^[^ ^68^ ^]^ Their reduced expression suggests that GM‐CAp may attenuate neutrophil recruitment to the infection site by limiting endothelial adhesion and subsequent transmigration into local tissues. Neutrophils, while essential for early immune defense, can also exert “off‐target” effects. They eliminate pathogens by generating toxic superoxide and its metabolites, releasing antimicrobial peptides and forming NETs.^[^ ^69^ ^]^ However, in the context of severe or recurrent SSIs, neutrophil disorder can occur, indicating that the problem lies in the number or function of neutrophils. This dysfunction may result in phenotype switching and excessive cytokine production, and it may contribute to fibrosis, tumorigenesis, or other pathological outcomes.^[^ ^70^, ^71^ ^]^ In addition, neutrophils can inhibit bone formation by directly contacting osteoblasts and suppressing osteogenic differentiation, while neutrophil extracellular traps (NETs) can mediate bone erosion in rheumatoid by enhancing RANKL‐induced osteoclastogenesis^[^ ^72^, ^73^ ^]^ Studies have shown that mitigating neutrophil infiltration can promote tissue recovery, such as the restoration of myocardial function following ischemic injury.^[^ ^74^ ^]^ On the basis of these observations and previous reports, we postulate that successful reduction of the bacterial count by GM‐CAp may primarily result from the direct antibacterial effect of gentamicin release, rather than from neutrophil accumulation and action. Therefore, in addition to bacterial quantification, which is recognized as the gold standard for evaluating antibacterial efficacy, the status of early postoperative neutrophil recruitment may serve as a potential parameter for evaluating whether antibacterial materials effectively interrupt inflammatory cascades. The presence or absence of neutrophil recruitment could also be correlated with the possibility of subsequent tissue regeneration, which is essential for preventing serious complications.

On‐site pharmacokinetics is usually monitored with microdialysis, which poses challenges for drug‐loaded biomaterials owing to its invasiveness and high operational complexity.^[^ ^75^ ^]^ In contrast, omics‐based enrichment analysis of drug metabolism pathways (e.g., cytochrome P450) provides indirect evidence of gentamicin bioavailability,^[^ ^76^ ^]^ offering a novel approach for assessing the in situ performance of drug‐loaded biomaterials.

Gentamicin has been incorporated into diverse carrier systems, including calcium sulfate,^[^ ^77^ ^]^ various calcium phosphate cements,^[^ ^78^ ^]^ and biphasic ceramic bone substitutes,^[^ ^79^ ^]^ for the prevention or treatment of osteomyelitis. However, most of these studies are focused on in vitro assessments (e.g., drug‐release kinetics and biofilm inhibition assays), with few addressing in vivo functional outcomes. In parallel, new antibacterial/osteogenic concepts have emerged, such as near‐infrared photothermal approaches aimed at repairing mitochondrial function and tea polyphenol‐modified scaffolds simulating natural bone tissues to endow dual functionality, although their translation remains to be established.^[^ ^80^, ^81^, ^82^ ^]^ In this study, GM‐CAp granules achieved multiple biological effects, including antibacterial and osteogenic effects. This synergistic multifunctionality enhances the success rate of CAp in repairing bone defects within a bacterial environment. However, this study has certain limitations. The potential influence of gentamicin release in the late stages of bone healing requires further investigation. Moreover, antibacterial evaluation in this study was focused on planktonic and inoculated bacteria, without addressing established biofilms. Further studies are required to examine GM‐CAp activity against mature biofilms on material surfaces. In addition, further long‐term in vivo studies are required to validate the sustained antibacterial performance and bone remodeling capacity of GM‐CAp. Finally, establishing an SSI model specifically for maxillofacial surgery is necessary to more accurately mimic the complex environment of bone defects in the oral cavity, which is characterized by a complex microbiota, saliva, and mechanical loading.

## 4 Conclusion

GM‐CAp granules were fabricated by physically precipitating gentamicin onto the surface of CAp granules. After implantation, GM‐CAp granules released gentamicin to the surgical site in the early postoperative stage, enabling the timely suppression of pathogenic bacterial growth. This early and effective antibacterial action mitigated neutrophil accumulation during the initial phase of bone reconstruction and facilitated subsequent bone regeneration, thereby enhancing the success rate of CAp in repairing bone defects within bacterial environments. This study demonstrates the potential of GM‐CAp granules for the treatment of SSIs associated with dental and orthopedic surgeries. Furthermore, it proposes the status of early postoperative neutrophil recruitment as a potential parameter for evaluating the antibacterial efficacy of biomaterials.

## 5 Experimental Section

### Fabrication of Gentamicin‐Released CAp Granules

CAp granules with diameters of 300–600 µm were kindly provided by GC Corporation (Tokyo, Japan). A gentamicin sulfate solution was prepared by dissolving gentamicin sulfate (Gibco, Grand Island, USA) in distilled water. In the preliminary experiment, the concentration of the gentamicin sulfate solution was adjusted to achieve a clinically relevant gentamicin release profile that balances antibacterial efficacy with biocompatibility by referring to the release characteristics of G‐HV. Then, gentamicin‐loaded carbonate apatite (GM‐CAp) granules were prepared by immersing the CAp granules in 200 mg mL^−1^ gentamicin solution for 24 h. After 24 h, the immersed granules were washed and lyophilized. Both CAp and GM‐CAp granules were sterilized with ethylene oxide for 24 h prior to use. TG‐DTA was conducted using a thermogravimetric analyzer (DTG‐60H, Shimadzu, Kyoto, Japan). In this analysis, CAp and GM‐CAp granules (10 mg) were separately heated up to 500 °C at a heating rate of 10 °C min^−1^ under air to determine the gentamicin content of the GM‐CAp granules.

### Determination of Gentamicin Release from GM‐CAp Granules

To assess gentamicin release, 100 mg of GM‐CAp granules were soaked in 1 mL of distilled water or PBS for 3, 24, 48, and 72 h. The eluent was collected, and the gentamicin concentration in the solution was determined using colorimetry. At each time point, 200 µL of the soaking solution was mixed with 40 µL of 2% NaHCO_3_ solution, followed by the addition of 2.5 µL of 2,4‐dinitrofluorobenzene (FDNB) in 95% ethanol. The mixture was incubated at 60 °C for 30 min, and then 20 µL of 1 m HCl was added. The optical density (OD) at 410 nm was measured. A standard curve was generated using standard solutions with gentamicin concentrations ranging from 25 to 250 µg mL^−1^. G‐HV served as a standard material to compare the gentamicin release profile of GM‐CAp granules.

### Measurement of MIC and MBC of Gentamicin Sulfate Against Oral Bacterial Species

Six odontogenic bacterial species, namely *S. aureus* ATCC29213 (American Type Culture Collection, Manassas, USA), *Streptococcus pyogene*s ATCC19615, *Streptococcus sanguinis* ST3R, *Streptococcus sobrinus* NCTC121279, *Fusobacterium nucleatum* 1436, and *Parvimonas micra* GIFU7745, were cultivated at 37 °C for 24 h under the specific culture conditions shown in **Table** **2**. The MIC and MBC of gentamicin sulfate against six bacterial species were measured with a microdilution assay. Briefly, a gentamicin sulfate solution was serially diluted from 1024 to 0.0625 µg mL^−1^. All bacterial strains were cultured to passage 2 and adjusted to 2 × 10^6^ CFU mL^−1^. Then, 1 µL of each gentamicin sulfate solution (0.0625–1024 µg mL^−1^) was added into each well of a 96‐well microplate, followed by 100 µL of each bacterial suspension with the corresponding broth, thus diluting the original solution 2 times. The plates were incubated at 37 °C for 24 h under specific culture conditions. Then, aliquots of 100 µL were taken from the wells and inoculated onto agar plates suitable for each species. After subculturing for 48 h, the MBC was determined as the lowest concentration at which no bacterial growth was observed.

**Table 2:** Bacteria and culture conditions.

Species	Broth	Agar	Culture condition
*Staphylococcus aureus* ATCC29213	Brain heart infusion broth	Brain heart infusion agar	Aerobic
*Streptococcus pyogenes* ATCC19615	Brain heart infusion broth	Brain heart infusion agar	Aerobic
*Streptococcus sanguinis* ST3R	Brain heart infusion broth	Brain heart infusion agar	Anaerobic
*Streptococcus sobrinus* NCTC121279	Brain heart infusion broth	Brain heart infusion agar	Anaerobic
*Fusobacterium nucleatum* 1436	Todd Hewitt broth containing 0.1% L‐cysteine	Todd Hewitt broth agar containing 0.1% L‐cysteine	Anaerobic
*Parvimonas micra* GIFU7745	Brain heart infusion broth	Brain heart infusion agar	Anaerobic

### In Vitro Effect of GM‐CAp Granules Against Odontogenic Bacteria: Agar Diffusion Tests

The suspension of each bacterial species was adjusted to ≈1 × 10^6^ CFU mL^−1^. Then, 200 µL of each suspension was separately spread onto an agar plate using a spiral plating system (Eddy Jet, IUL, Barcelona, Spain). Subsequently, 100 mg of either CAp or GM‐CAp granules was placed into a well (diameter = 9 mm) prepared in each agar plate. All plates were incubated at 37 °C for 48 h. The effect of each material on the bacteria was assessed by confirming the presence or absence of inhibition zones. Each test was repeated three times.

### In Vitro Effect of GM‐CAp Granules Against Odontogenic Bacteria: Bacterial Growth in the Presence of CAp and GM‐CAp Granules

Bacterial suspensions of the 6 species were adjusted to ≈1 × 10^6^ CFU mL^−1^. Then, 1 mL of each bacterial suspension was mixed with 50 mg of CAp or GM‐CAp granules and incubated for 3 and 24 h (*n* = 3). After incubation, visible bacteria were quantified using the spread plate method. Briefly, granules were removed using a 10 µm pore size filter, and 100 µL of the bacterial suspension was diluted with 9.9 mL of each medium (Table 2). Then, each diluted suspension was serially diluted and incubated on an agar plate. Subsequently, the plates were incubated at 37 °C for 24 h, and the colonies formed were counted.

### In Vitro Evaluation of Biocompatibility: Cell Culture

Human bone marrow‐derived MSCs (PT‐2501, Lonza, Walkersville, USA) were cultured in minimum essential medium α (MEMα, Wako, Tokyo, Japan) supplemented with 20% fetal bovine serum (Nichirei Biosciences, Tokyo, Japan) and 1% penicillin/streptomycin (Sigma–Aldrich, St. Louis, USA) in a 5% CO_2_ incubator at 37 °C. Cells were expanded and used between passages 3–5 for experiments.

### In Vitro Evaluation of Biocompatibility: Cytotoxicity

To prepare eluates from the materials, CAp or GM‐CAp granules were soaked in 5 mL of complete medium and shaken at 10 rpm and 37 °C for 24 h. The granules were then removed by filtration (0.45 µm pore size) to obtain the eluate (100%). The eluate was serially diluted 2‐fold with complete medium to four concentrations (50%, 25%, 12.5%, and 6.25%) to prepare the conditioned medium. MSCs were seeded at a density of 1.0 × 10^4^ cells per well in 96‐well plates. After 24 h, the medium was replaced with 100 µL of the conditioned medium (100%, 50%, 25%, 12.5%, and 6.25% of the eluate). Cells treated with 100% eluate from CAp granules served as the control group. After 24 h, cell viability was assessed using the WST assay (Cell Counting Kit‐8, CCK‐8, Dojindo, Mashiki, Japan). Briefly, cells were washed twice with phosphate buffered saline (PBS) and incubated with fresh medium containing 10% CCK‐8 solution at 37 °C for 3 h. The OD at 450 nm was measured using a microplate reader (iMark, Bio‐Rad Laboratories, Hercules, USA) (*n* = 4).

### In Vitro Evaluation of Biocompatibility: Dose Response

To optimize the treatment dose of the granules, a dose‐response assay was performed. MSCs were seeded in 24‐well plates at a density of 1.0 × 10^4^ cells per well. Cells were then treated with different doses (2, 5, 10, and 20 mg) of CAp or GM‐CAp granules placed in cell culture inserts (8.0 µm pore size, FALCON, Corning, USA). After 7 days of incubation, cell viability was assessed using a CCK‐8 kit (*n* = 4). Cells without exposure to CAp or GM‐CAp granules were used as the control.

### In Vitro Evaluation of Biocompatibility: Cell Proliferation

To evaluate the influence of gentamicin release on cell proliferation, MSCs were treated with 10 mg of CAp or GM‐CAp granules placed in cell culture inserts in 24‐well plates for 1, 3, and 7 days. Cells without exposure to CAp or GM‐CAp granules were used as the control. Cell viability was assessed using a CCK‐8 kit (*n* = 4).

### In Vitro Evaluation of Biocompatibility: Osteogenic Differentiation

To assess the influence of gentamicin release on osteogenic differentiation, ALP activity was determined using a TRACP & ALP assay kit (Takara Bio, Kusatsu, Japan). MSCs were treated with 10 mg of CAp or GM‐CAp granules for 7 and 14 days. Cells without exposure to CAp or GM‐CAp granules were used as the control. Cell lysates were extracted with an extraction solution, then mixed with 50 µL of the substrate solution, and incubated at 37 °C for 60 min. The reaction was terminated by adding 50 µL of the stop solution to each well. The absorbance at 405 nm was measured using a microplate reader (iMark). ALP activity was normalized to the protein concentration determined using a Bio‐Rad protein assay (Bio‐Rad Laboratories) (*n* = 4).

Osteogenic gene expression of MSCs was also investigated using the real‐time polymerase chain reaction after 3, 7, and 14 days of culturing. Gene expression of *Runx2*, *Alp*, *Opg*, and glyceraldehyde 3‐phosphate dehydrogenase (*Gapdh*) was quantified using a cell‐to‐Ct kit (ThermoFisher Scientific, Waltham, USA) (*n* = 4). The relative expression of *Runx2*, *Alp*, and *Opg* to that of *Gapdh* was calculated using the 2^−∆∆Ct^ method. The results of the control after 3 days of culturing was used to normalize all results.

### In Vivo Antibacterial Effect Evaluation: Bacterial Strain

A fresh colony of *S. aureus* ATCC29213 was inoculated into brain heart infusion (BHI) broth and incubated at 37 °C overnight. The culture was then diluted 1:50 with BHI broth and incubated at 37 °C for 4 h. Bacteria were harvested by centrifugation and resuspended in PBS. The bacterial density was determined by measuring the absorbance at 600 nm (A_600_) using a spectrophotometer (BioPhotometer Plus, Eppendorf, Hamburg, Germany). The bacterial suspension was diluted with PBS to a concentration of 1.0 × 10^7^ CFU mL^−1^ on the basis of a standard growth curve to determine the bacterial concentration from the A_600_ value.

### In Vivo Antibacterial Effect Evaluation: Murine model of SSIs

All animal handling and treatment protocols were approved by the Institutional Animal Care and Use Committee of The University of Osaka, Graduate School of Dentistry (Approval No. R‐04‐006‐0).

A schematic of the design of the murine model of infected bone defects representing SSIs is shown in Figure 3a. Eleven‐week‐old male C57BL/6JJcl mice (CLEA Japan, Tokyo, Japan) were anesthetized by isoflurane inhalation and intraperitoneally injected with a mixture of butorphanol (2.5 µg g^−1^), midazolam (2.0 µg g^−1^), and medetomidine (0.375 µg g^−1^) on the basis of the weight of each mouse. A cylindrical cavity with a diameter of 1.0 mm and depth of 1.5 mm was created in the left femoral condyle of each mouse using a 0.8 mm fissure bur (Dentsply Sirona, Charlotte, USA). The bone cavity was thoroughly washed with sterile saline to remove bone debris. Subsequently, 1.2 µL of the prepared *S. aureus* suspension (≈10^4^ CFU of *S. aureus*) was inoculated in each bone cavity. The cavity was then filled with 1.5 mg of either CAp or GM‐CAp granules (*n* = 6), and the wound was closed. The survival rate was monitored daily. At 3 and 14 days post‐surgery, the mice were euthanized by cervical dislocation, and the surgical sites were photographed.

### In Vivo Antibacterial Effect Evaluation: CFU Enumeration in Bone Tissue

Femurs were harvested for CFU enumeration. The surrounding soft tissue was gently removed, and the femurs were cut into small pieces using a dissecting scissor. The pieces were homogenized in 1 mL of BHI broth by vortexing at 3000 rpm for 15 min. Homogenates containing *S. aureus* were serially diluted and plated onto BHI agar plates using a spiral plating system. Plates were incubated at 37 °C under aerobic conditions for 24 h, and the colonies were counted to determine the total CFU.

### In Vivo Antibacterial Effect Evaluation: Micro‐CT

Harvested femurs were fixed in 10% formalin‐buffered solution for 24 h. Bone destruction was analyzed using micro‐CT (R_mCT2, Rigaku, Tokyo, Japan) with a scan field‐of‐view of 20 × 20 mm and resolution of 40 µm. Bone areas were identified according to the CT values and the phantom control of hydroxyapatite (200–800 mg cm^−3^). The volume ratio of regenerated bone to tissue volume within the defect area was measured using bone‐specific image analysis software (TRI/3D‐BON, RATOC, Tokyo, Japan).

### In Vivo Antibacterial Effect Evaluation: Tissue Preparation and Histopathological Staining

Following micro‐CT analysis, fixed femurs were decalcified in Kalkitox solution (Wako) for 6 days. Specimens were embedded in paraffin using an automatic paraffin embedding device (CT‐Pro20, Genostaff, Tokyo, Japan) and cut into 5 µm‐thick sections using a microtome (2125RT, Leica, Nussloch, Germany). To investigate wound healing and osteogenesis, sections were stained with H&E and Masson–Goldner's trichrome (Sigma–Aldrich). To visualize bacteria infection and immune cell infiltration, sections were stained with a Gram stain (modified Brown & Brenn, ScyTek Laboratories, Logan, USA) and naphthol AS‐D CAE stain (Muto Pure Chemicals, Tokyo, Japan) according to the manufacturer's instructions. Stained sections were examined under a light microscope (ECLIPSE Ci‐L, Nikon, Tokyo, Japan). The AS‐D CAE stained area relative to the total defect area was determined using Image J.

### In Vivo Antibacterial Effect Evaluation: Hematological Analysis

Peripheral blood was collected from the submandibular vein of each mouse at 3 and 14 days post‐surgery. Blood samples were stored in EDTA‐preconditioned tubes (Microtainer, Becton, Dickinson and Company, Franklin Lakes, USA) at 4 °C for subsequent hematological analysis. Hematological analysis was performed by Biosafety Research Center Inc. (Kobe Research Institute, Kobe, Japan). Total WBC counts and percentages of specific types of WBCs, namely neutrophils, monocytes, lymphocytes, eosinophils, and basophils, were measured at two time points. Blood samples from age‐matched mice without surgery served as the untreated control. The control group was composed of age‐matched healthy mice without surgery, bacterial inoculation, or material implantation.

### In Vivo Antibacterial Effect Evaluation: Transcriptomic Sequencing

Transcriptomic sequencing and bioinformatics analysis were conducted to explore the patterns of early innate immune response to SSIs. Nine mice were randomly divided into three groups: two material groups (CAp and GM‐CAp) and one without‐surgery group (control). The control group was composed of age‐matched healthy mice without surgery, bacterial inoculation, or material implantation. On day 3, total RNA was extracted from the femoral condyle using a NucleoSpin RNA Plus XS kit (Takara) according to the manufacturer's instructions.

Sample RNA content, integrity, and purity were evaluated using Nanodrop 2000 (Thermo Fisher Scientific), agarose gel electrophoresis (Thermo Fisher Scientific), and Agilent 5400 Fragment Analyzer (Agilent, Santa Clara, USA). Sequencing was performed using a high‐throughput sequencing platform, DNBSEQ‐T7 (MGI Tech Co., Shenzhen, China). Differential expression was analyzed using the DESeq2 R package (1.42.0). The resulting *p‐*value was adjusted using Benjamini and Hochberg's methods to control the error discovery rate. The threshold of significant differential expression was *padj* <= 0.05 & |log2(foldchange)| >= 1. GO enrichment analysis of differentially expressed genes was performed using the clusterProfiler R package (4.8.1), in which the gene length bias was corrected. GO terms with *padj*‐value < 0.05 were considered significantly enriched by differentially expressed genes. KEGG pathway enrichment analysis on the set of differential genes was conducted using clusterProfiler. KEGG pathways with a *p*‐value < 0.05 were considered significantly enriched.

### Statistical Analysis

Results are presented as mean ± standard deviation. Student's *t*‐test was used to compare means between two groups. The non‐parametric Kruskal–Wallis test, followed by Dunn's multiple comparison test, was performed to compare hematological data among control, CAp, and GM‐Cap groups. In addition to the *p*‐value, 95% confidence intervals were calculated to estimate the precision. A *p*‐value <0.05 was considered statistically significant.

## Conflict of Interest

The authors declare no conflict of interest.

## Author Contributions

L.X. wrote original draft, performed data curation, formal analysis, funding acquisition, investigation, methodology, visualization; G.L.A. performed formal analysis, investigation, methodology; J.‐I. S. wrote, reviewed, and edited the manuscript, performed methodology, supervision; H.K. performed supervision, methodology, wrote, reviewed, and edited the manuscript; R.T. performed data curation, methodology; T.K. performed data curation, formal analysis, methodology; S.I. performed conceptualization, supervision, project administration, and acquired resources.

## Supporting information



Supporting Information

## Data Availability

The data that support the findings of this study are available from the corresponding author upon reasonable request.
