# Supplementary material for: Gentamicin‐Loaded Carbonate Apatite with Dual Antibacterial and Osteogenic Functions for Combating Surgical Site Infections
Source: Adv Healthc Mater. 2025 Oct 26;15(8):e03739. doi: 10.1002/adhm.202503739 (PMC12927543; doi:10.1002/adhm.202503739)
Supplement: Supplementary file 1 — Supporting Information [file ADHM-15-0-s001.docx]

Supporting Information

Gentamicin-loaded carbonate apatite with dual antibacterial and osteogenic functions for combating surgical site infections

Linghao Xiao*, Gabriela Laranjeira Abe, Jun-Ichi Sasaki, Haruaki Kitagawa, Ririko Tsuboi, Tomoki Kohno, Satoshi Imazato

L. Xiao, G. L. Abe, J.-I. Sasaki, H. Kitagawa, T. Kohno, S. Imazato

Joint Research Laboratory of Advanced Functional Materials Science, The University of Osaka, Graduate School of Dentistry, 1-8 Yamadaoka, Suita, Osaka 565-0871, Japan.

E-mail: xiao.linghao.dent@osaka-u.ac.jp (L. Xiao)

J.-I. Sasaki, H. Kitagawa, S. Imazato
Department of Dental Biomaterials, The University of Osaka, Graduate School of Dentistry, 1-8 Yamada-Oka, Suita, Osaka 565-0871, Japan.

R. Tsuboi

Department of Cariology, Restorative Sciences and Endodontics, University of Michigan School of Dentistry, 1011 N University Avenue, Ann Arbor, MI 48104, USA.


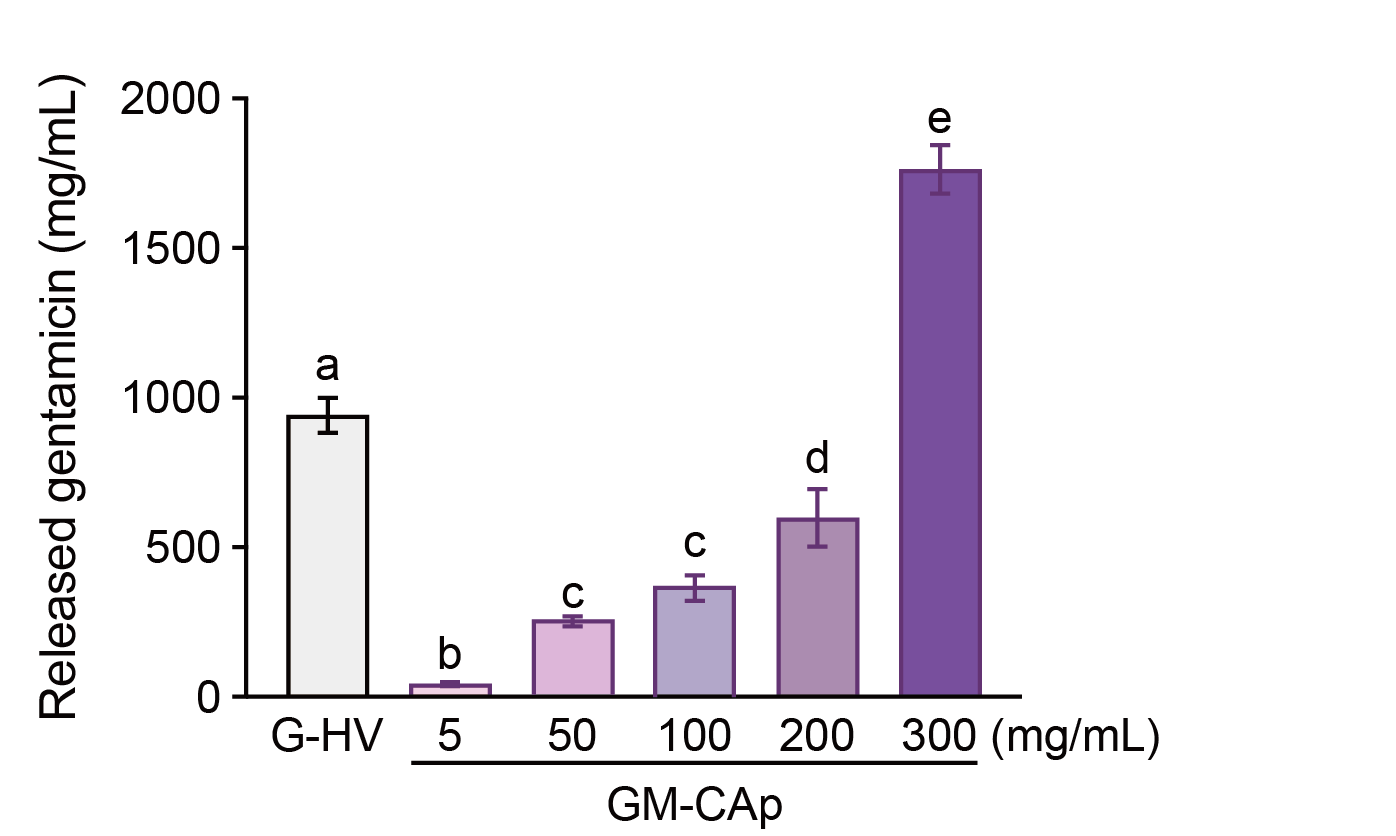


**Figure S1.** Release of gentamicin from G-HV and GM-CAp granules prepared under different conditions (*n* = 3). Different letters indicate significant statistical differences (*p* < 0.01) (*n* = 3).


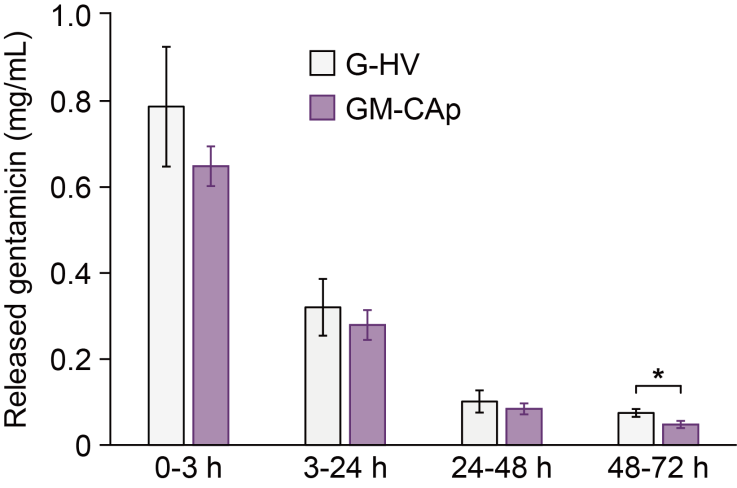


**Figure S2.** Release of gentamicin from G-HV and GM-CAp granules (*n* = 3). * *p* < 0.05.

**
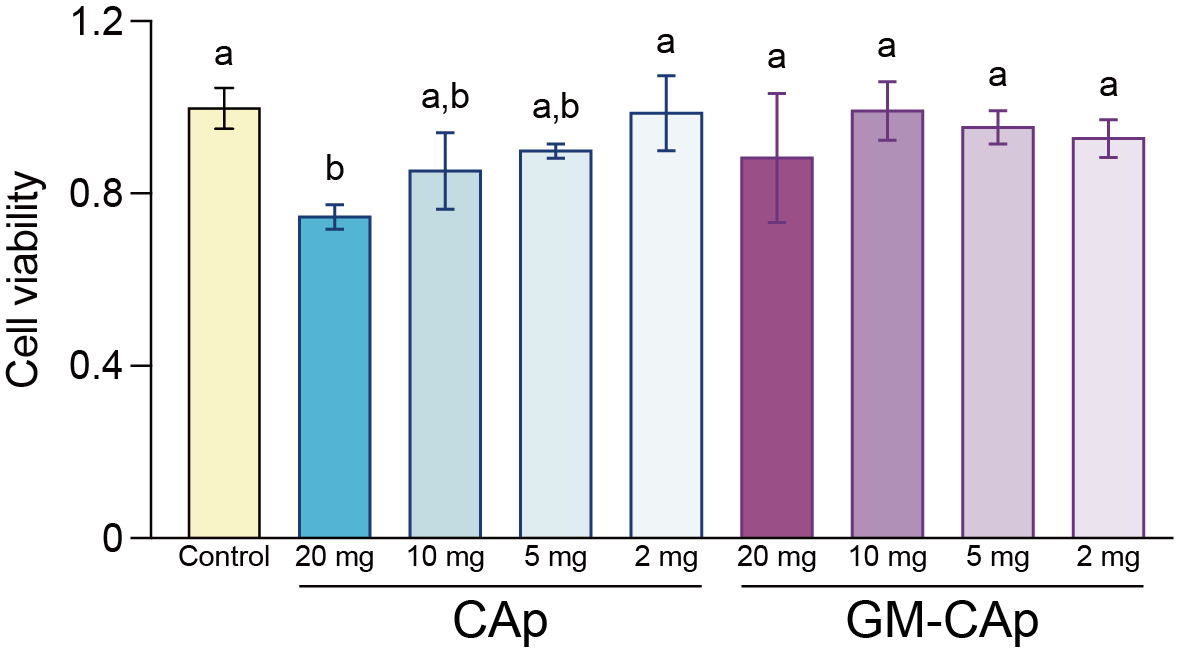
**

**Figure S3.** Response of MSCs to different doses of CAp and GM-CAp granules after 7 days of culturing (*n* = 4). Different letters indicate significant statistical differences (*p* < 0.01).


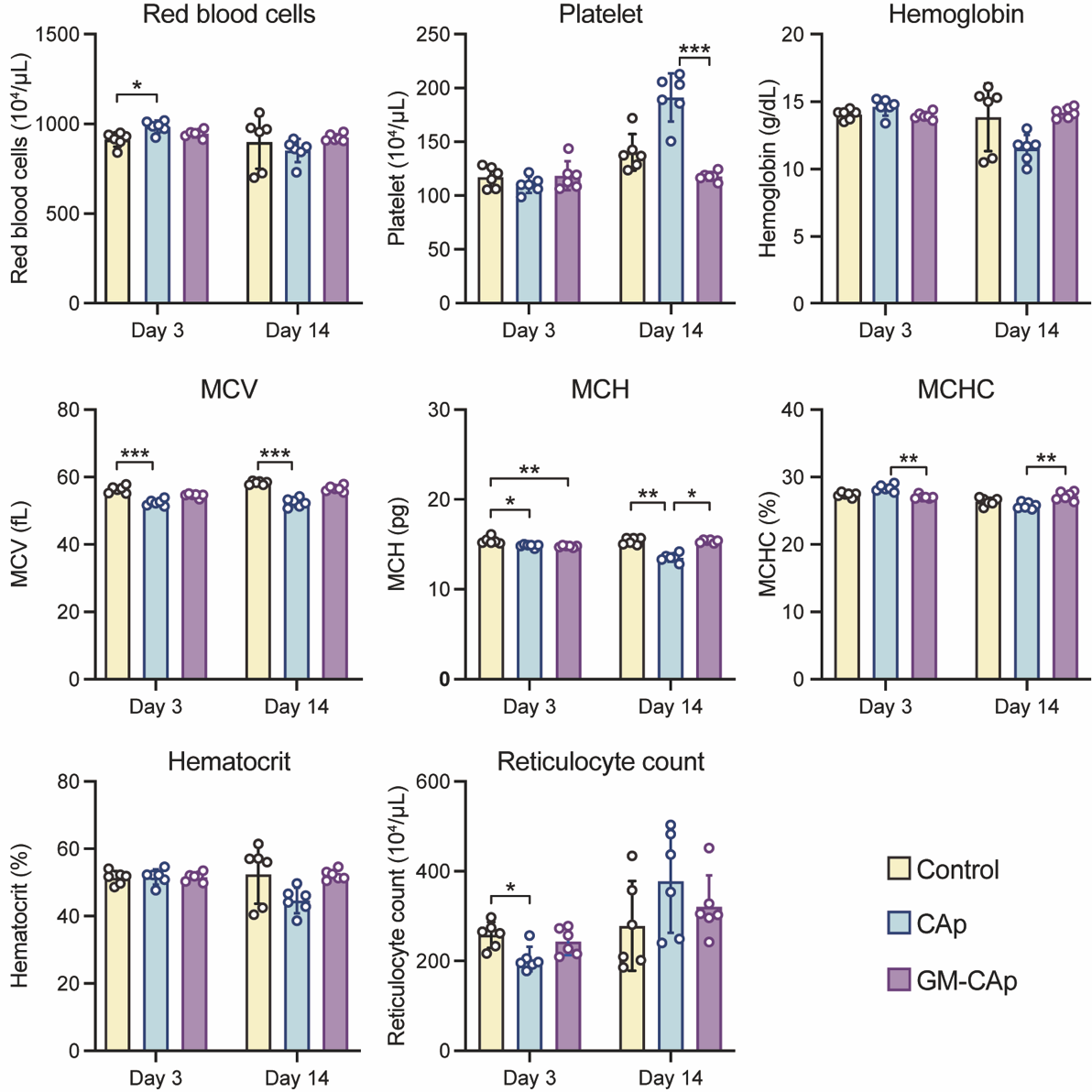


**Figure S4.** Hematological analysis at 3 and 14 days post-surgery to evaluate red blood cells, platelet, hemoglobin, mean corpuscular volume (MCV), mean corpuscular hemoglobin (MCH), mean corpuscular hemoglobin concentration (MCHC), hematocrit, and reticulocyte count (*n* = 6). * *p* < 0.05, ** *p* < 0.01, *** *p* < 0.001.


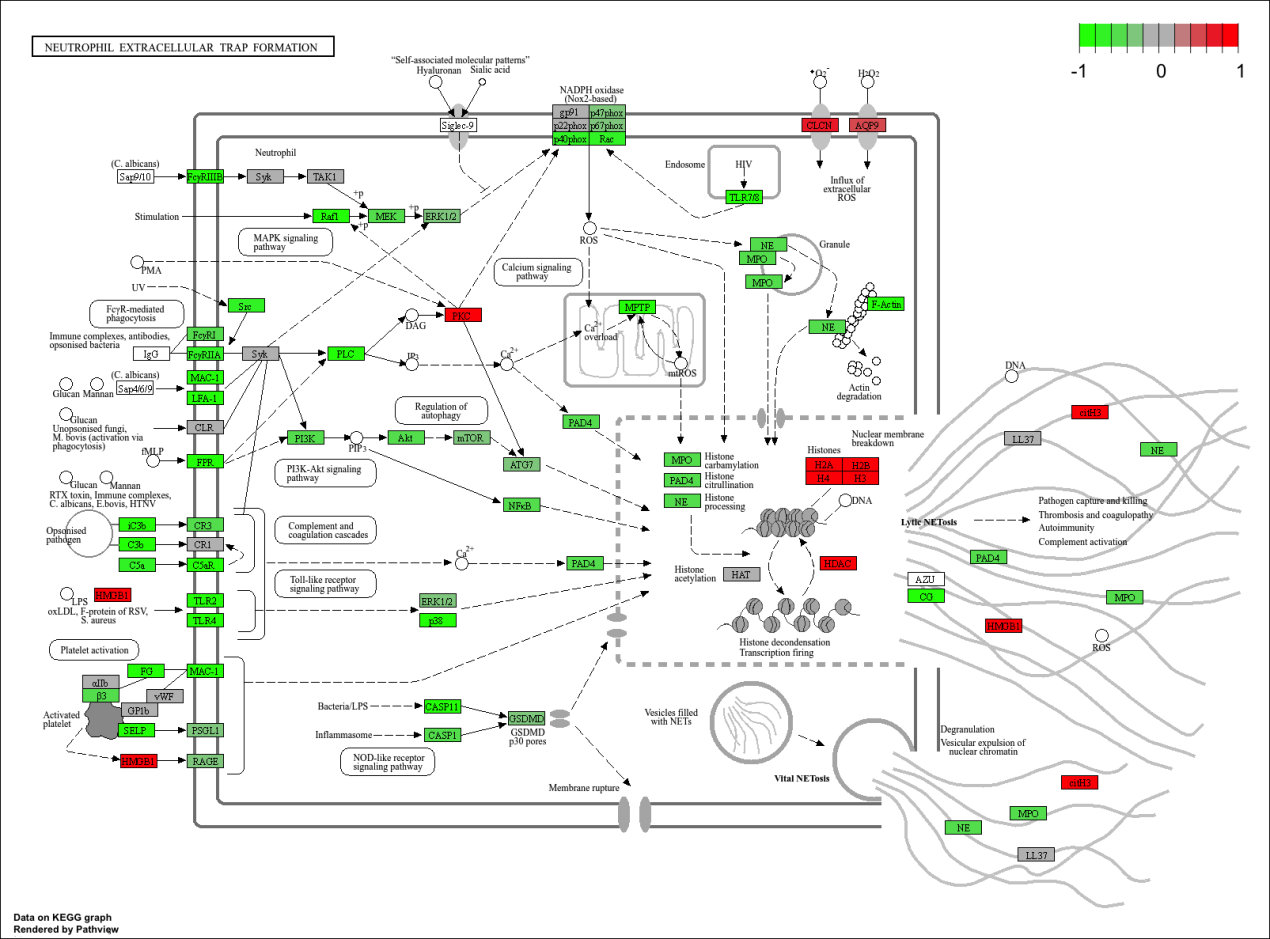


**Figure S5.** KEGG pathway map of neutrophil extracellular trap formation signaling pathway. Green and red boxes indicate downregulated and upregulated genes, respectively.

**Table S1.** Clinical symptoms of each sample at two time points.

| Group | Time point | Animal No. | Swelling | Redness | Abscess | Dysfunction |
| --- | --- | --- | --- | --- | --- | --- |
| CAp | Day 3 | 1 | Severe | Moderate | Small | Severe |
|  |  | 2 | Severe | Moderate | Small | Severe |
|  |  | 3 | Moderate | Moderate | Small | Severe |
|  |  | 4 | Mild | Mild | Small | Severe |
|  |  | 5 | Mild | Mild | None | Severe |
|  |  | 6 | Severe | Mild | Small | Severe |
|  | Day 14 | 1 | Severe | None | Large | Moderate |
|  |  | 2 | Severe | None | Large | Moderate |
|  |  | 3 | Severe | None | Large | Moderate |
|  |  | 4 | Severe | None | Large | Moderate |
|  |  | 5 | Severe | None | Large | Moderate |
|  |  | 6 | Severe | None | Large | Moderate |
| GM-CAp | Day 3 | 1 | Mild | Mild | None | Moderate |
|  |  | 2 | Moderate | Mild | None | Moderate |
|  |  | 3 | Moderate | Mild | Small | Moderate |
|  |  | 4 | Mild | Mild | None | Moderate |
|  |  | 5 | Mild | Mild | None | Moderate |
|  |  | 6 | Moderate | Mild | None | Moderate |
|  | Day 14 | 1 | None | None | None | None |
|  |  | 2 | None | None | None | None |
|  |  | 3 | None | None | None | None |
|  |  | 4 | None | None | None | None |
|  |  | 5 | None | None | None | None |
|  |  | 6 | None | None | None | None |

**Table S2.** 95% confidence intervals (CIs) for hematological parameters.

| Parameter | Period | Group | Mean | 95% CI of mean (Lower–Upper) |
| --- | --- | --- | --- | --- |
| WBCs (× 10^3^ /µL) | Day 3 | Control | 7.216 | 6.107–8.326 |
|  |  | CAp | 5.550 | 4.870–6.229 |
|  |  | GM-CAp | 6.150 | 5.376–6.923 |
|  | Day 14 | Control | 7.700 | 6.380–9.019 |
|  |  | CAp | 5.766 | 4.693–6.840 |
|  |  | GM-CAp | 6.766 | 6.028–7.504 |
| Monocyte/WBCs (%) | Day 3 | Control | 1.683 | 1.063–2.304 |
|  |  | CAp | 0.683 | 0.2997–1.067 |
|  |  | GM-CAp | 1.716 | 1.3053–2.128 |
|  | Day 14 | Control | 2.033 | 0.6275–3.4392 |
|  |  | CAp | 4.333 | 3.712–4.955 |
|  |  | GM-CAp | 1.083 | 0.7054–1.4612 |
| Neutrophils/WBCs (%) | Day 3 | Control | 10.80 | 8.518–13.082 |
|  |  | CAp | 30.63 | 23.197–38.068 |
|  |  | GM-CAp | 18.00 | 11.407–24.593 |
|  | Day 14 | Control | 20.818 | 4.494–37.139 |
|  |  | CAp | 42.166 | 35.178–49.154 |
|  |  | GM-CAp | 9.566 | 8.921–10.212 |
| Lymphocytes/WBCs (%) | Day 3 | Control | 84.866 | 81.661–88.0723 |
|  |  | CAp | 66.017 | 59.121–72.912 |
|  |  | GM-CAp | 77.5 | 70.539–84.461 |
|  | Day 14 | Control | 75.2 | 58.245–92.156 |
|  |  | CAp | 51.267 | 44.797–57.736 |
|  |  | GM-CAp | 86.867 | 85.809–87.923 |
| Eosinophils/WBCs (%) | Day 3 | Control | 2.483 | 1.612–3.355 |
|  |  | CAp | 2.5167 | 0.7956–4.2387 |
|  |  | GM-CAp | 2.55 | 2.007–3.092 |
|  | Day 14 | Control | 1.75 | 0.887–2.612 |
|  |  | CAp | 2.083 | 1.224–2.942 |
|  |  | GM-CAp | 2.133 | 1.754–2.512 |
| Basophils/WBCs (%) | Day 3 | Control | 0.15 | 0.0925–0.2075 |
|  |  | CAp | 0.1833 | 0.1043–0.2623 |
|  |  | GM-CAp | 0.2 | 0.1061–0.2939 |
|  | Day 14 | Control | 0.1667 | 0.1125–0.2209 |
|  |  | CAp | 0.1167 | 0.0377–0.1957 |
|  |  | GM-CAp | 0.3667 | 0.1713–0.5621 |
